# Enhancing Neuroplasticity in the Chronic Phase After Stroke: Effects of a Soft Robotic Exosuit on Training Intensity and Brain-Derived Neurotrophic Factor

**DOI:** 10.1109/OJEMB.2023.3313396

**Published:** 2023-09-08

**Authors:** Anna V. Roto Cataldo, Ashley N. Collimore, Johanna Spangler, Lillian Ribeirinha-Braga, Karen Hutchinson, Qing Mei Wang, LaDora Thompson, Louis N. Awad

**Affiliations:** ^1^ Boston University, College of Health and Rehabilitation Sciences: Sargent 1846 Boston MA 02215 USA; ^2^ Spaulding Rehabilitation Hospital 24498 Charlestown MA 02129 USA

**Keywords:** Brain-derived neurotrophic factor, neuroplasticity, soft robotic exosuit, stroke, walking

## Abstract

*Objective:* High intensity training may enhance neuroplasticity after stroke; however, gait deficits limit the ability to achieve and sustain high walking training intensities. We hypothesize that soft robotic exosuits can facilitate speed-based gait training at higher intensities and longer durations, resulting in a corresponding increase in circulating brain-derived neurotrophic factor (BDNF). *Results:* Eleven individuals >6-mo post-stroke completed a two-session, pilot randomized crossover trial (NCT05138016). Maximum training speed (Δ: 0.07 ± 0.03 m/s), duration (Δ: 2.07 ± 0.88 min), and intensity (VO_2_ peak, Δ: 1.75 ± 0.60 ml-O_2_/kg/min) significantly increased (p < 0.05) during exosuit-augmented training compared to no-exosuit training. Post-session increases in BDNF (Δ: 5.96 ± 2.27 ng/ml, p = 0.03) were observed only after exosuit-augmented training. Biomechanical changes were not observed after exosuit-augmented training; however, a deterioration in gait propulsion symmetry (%Δ: −5 ± 2 %) and an increase in nonparetic propulsion (Δ: 0.9 ± 0.3 %bw) were observed (p < 0.05) after no-exosuit training. *Conclusion:* Soft robotic exosuits facilitate faster, longer duration, and higher intensity walking training associated with enhanced neuroplasticity.

## I. Introduction

High intensity aerobic exercise has potential to enhance neuro-plasticity in the chronic phase of stroke recovery [1]. Indeed, in neurologically-intact individuals, acute high intensity exercise enhances motor skill acquisition [2], accuracy [3], and retention [4], [5], [6]. For example, motor learning was improved when a single bout of high intensity exercise either preceded or followed upper extremity motor task practice [5], [6], [7]. Similarly, in people with chronic stroke, motor skill retention improved when upper extremity training was completed immediately after [4] or before [8] intensive exercise. Though studies of lower extremity motor learning have been more mixed [9], the potential for high intensity aerobic exercise to prime the nervous system for motor learning is exciting [10].

A substantial amount of preclinical work [11], [12], [13] describes brain-derived neurotrophic factor (BDNF) as a likely key player in the beneficial priming effect intensive exercise has on learning. BDNF is a recognized biomarker of neuroplasticity, neurogenesis, and neuroprotection [14], [15], and is crucial in the adaptive responses of the brain and body to changes in energy balance, such as the metabolic shifts caused by high intensity exercise [16]. Indeed, the release of BDNF is dependent on exercise intensity [17], [18], [19], with exercise-induced increases in BDNF correlated with improvements in cognitive [13] and motor function [6] in both neurologically-intact humans and animal models of stroke [12], [20].

Post-stroke interventions that harness the neurobiological benefits of intensive exercise have the potential to enhance motor recovery; however, post-stroke neuromotor impairments that limit a patient's ability to achieve and maintain higher exercise intensities must be addressed. The majority of stroke survivors experience considerable and lasting gait deficits [21], [22] that contribute to compensatory movement strategies, gait asymmetries, and metabolically-expensive walking patterns [23], [24]. These deficits are associated with slow speeds, high fall risk, and high energetic costs [22], [25], [26], [27], [28], all walking characteristics disadvantageous toward maintaining high intensity walking for long durations. By limiting the ability to achieve and sustain fast walking after stroke, gait deficits indirectly reduce the overall energetic stimulus elicited by walking training and, in turn, reduce BDNF release into circulation. That is, stroke-induced neuromotor impairments that limit how fast and long people post-stroke can walk will naturally limit the intensity and duration of walking training.

**Figure 1.**
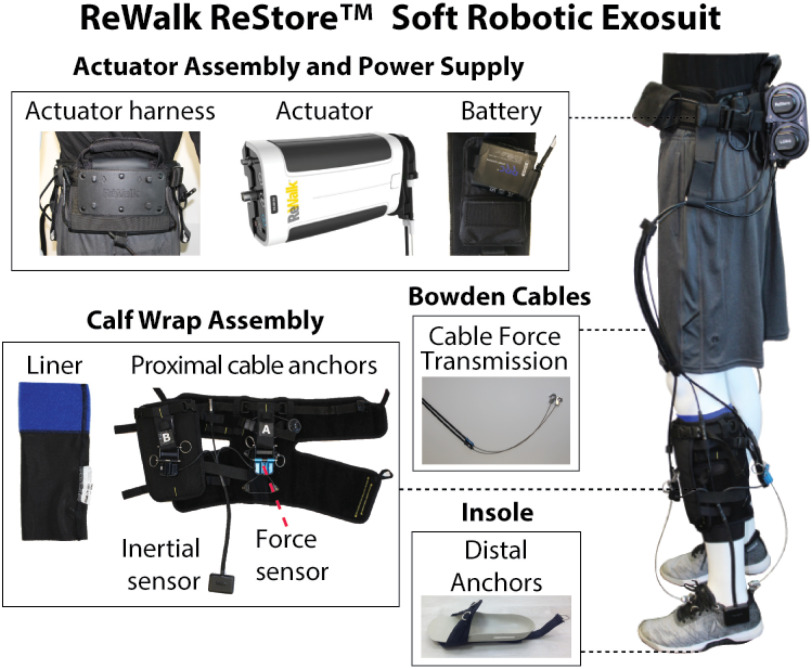
Soft Robotic Exosuit, ReWalk ReStore suit components. The ReStore is a textile-based exosuit that provides mechanical assistance to the paretic plantarflexors and dorsiflexors during walking.

Our team has developed a gait rehabilitation program that applies soft robotic exosuits to help people post-stroke achieve faster walking speeds and sustain them for longer durations [29]. More specifically, exosuits are textile-based devices (Fig. 1) that use cable-based force transmission to apply assistive joint torques in parallel with the underlying paretic muscles [30]. In prior work we show that exosuits improve gait mechanics and energetic efficiency [31] and reduce gait compensations [32] during comfortable-speed treadmill walking. Moreover, people post-stroke self-select faster overground walking speeds and walk farther distances while wearing the exosuit, compared to not wearing the exosuit [33]. Soft robotic exosuits have emerging promise as a tool for facilitating high-repetition, intensive, and task-specific gait training—training parameters known to drive experience-dependent neuroplasticity [34].

**Figure 2.**
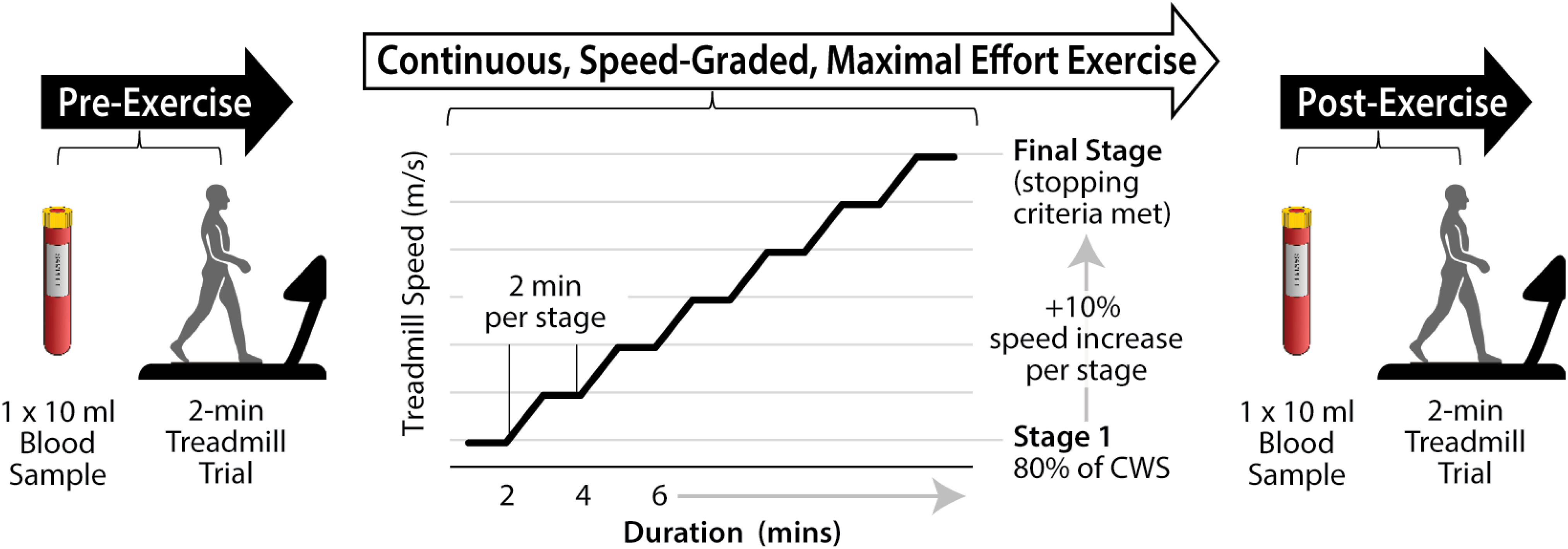
Session overview. Immediately before and after a continuous, speed-graded, maximal effort exercise bout, 10 ml blood samples were collected to enable comparative measurements of baseline and post-exercise serum BDNF levels. Before and after the exercise bout, subjects also completed a 2-minute biomechanical evaluation on an instrumented treadmill (without exosuit assistance) at their comfortable walking speed (CWS). The measured ground reaction force data were used to investigate pre-to-post training changes in interlimb peak propulsion symmetry. Indirect calorimetry and electrocardiogram data were collected *during* each exercise bout to determine between-condition differences in peak oxygen consumption (VO_2_ peak) and monitor subjects, respectively.

The objective of this foundational study was to evaluate the potential for harnessing the immediate changes in walking ability produced by soft robotic exosuits to facilitate gait training at higher intensities and for longer durations than is achieved without exosuits, thus promoting bioenergetic changes known to drive neuroplasticity. We recruited sixteen individuals in the chronic phase after stroke to participate in a pilot, randomized crossover trial consisting of two walking training sessions, one with and one without a soft robotic exosuit (see *Supplementary Figure 1, Study Schematic*). Each training session consisted of continuous, speed-graded, maximal effort walking exercise that spanned each subject's self-selected speed range and the maximum walking speeds that they could safely tolerate (Fig. 2). For each condition, we measured training intensity, duration, pre- and post-training circulating BDNF, and pre- and post-training gait symmetry. We hypothesized that the propulsive and ground clearance support provided to the paretic limb by soft robotic exosuits would facilitate faster walking speeds and minimize gait instabilities at faster speeds, ultimately resulting in a higher aerobic training intensity, measured by VO_2_ peak, and longer training duration. We also hypothesized that exosuit-induced increases in aerobic intensity would result in significantly higher circulating BDNF after versus before the training session, and larger improvements in gait ability, measured by improved interlimb propulsion symmetry.

## II. Results

### A. Participants and Data

In brief, sixteen individuals in the chronic phase of stroke recovery met initial study entry criteria and were further screened for eligibility, including adequate sizing and usability of the soft robotic exosuit (ReWalk Robotics, ReStore; Fig. 1). Of these sixteen, three were not eligible; one was not cleared by their physician and the two others could not be properly fit to an exosuit. Of the thirteen eligible individuals, two were not able to complete the study due to scheduling challenges. Eleven study participants were ultimately enrolled and randomized (see *Supplementary Figure 1: Study Schematic*). Study participants were an average 58 ± 13 years old, three were female, and seven had left-sided hemiparesis. Their average comfortable and maximum overground walking speeds were 0.90 ± 0.29 and 1.22 ± 0.54 m/s, respectively (Table 1).

**TABLE 1:** Participant Characteristics

Participant	Sex	Age	Paretic Side	Stroke Chronicity	Comfortable Over ground Walking Speed	Maximum Over ground Walking Speed	Age-Predicted Maximum Heart rate	Use of heart rate altering medication(s)	Use of Serotonergic Agents
	(M/F)	(y)	(Left/	(y)	(m/s)	(m/s)	(bpm)	(Y/N)	(Y/N)
			Right)						
1	M	49	Left	16.5	1.13	1.86	174	N	N
2	F	52	Left	7.25	1.04	1.14	172	N	N
3	M	48	Left	3.5	0.83	0.9	175	Y	N
4	M	62	Right	2.7	0.47	0.57	165	N	N
5	F	76	Left	–	0.54	0.64	156	Y	Y
6	M	47	Left	–	0.6	0.68	175	Y	N
7	M	80	Left	10.75	1.07	1.17	153	N	N
8	M	57	Left	10.25	1.08	1.57	169	N	Y
9	M	38	Right	9	1.31	2.14	181	N	N
10	M	65	Right	6.6	0.69	0.94	163	N	Y
11	F	61	Right	–	1.18	1.78	166	Y	N
Average (standard deviation)	27% F	58 (13)	64% Left sided	8 (4)	0.90 (0.29)	1.22 (0.54)	168 (9)	36% taking	27% taking

M = male; F = female; y = years; m/s = meters per second; bpm = beats per minute; Y = yes; N = no

The no-exosuit and exosuit-augmented training sessions were completed in random order across study participants and separated by a washout period that was at least 1 week, and no more than 2 weeks, in duration. Study data were collected before, during, or after each training session (see Fig. 2). Blood samples were collected before and after each training session and analyzed (Human BDNF Quantikine ELISA Kit: R&D Systems) to evaluate training-induced changes in serum BDNF (ng/mL) within each condition. Similarly, ground reaction force data (N) were collected before and after each training session via 2-minute treadmill trials (Bertec, Columbus, OH, USA). Interlimb propulsion symmetry was computed from the mass-normalized peaks of the anterior ground reaction force data recorded for each limb [35] and used to evaluate training-induced changes in gait symmetry within each condition.

During the training, indirect calorimetry data (Cosmed© K5, Rome, Italy) were collected and used to evaluate between-condition differences in peak oxygen consumption (ml-O_2_/kg/min; VO_2_ peak). The reasons for stopping the training, and the training time completed, were also logged for each subject and compared between conditions. One subject was not able to safely provide the blood samples required for the BDNF analyses. Complete datasets were available for all other analyses.

### B. Between-Session Differences in Training Progression

Study participants achieved a faster maximum walking speed (Δ: 0.07 ± 0.03 m/s, p = 0.04) and more training stages (Δ: 1.05 ± 0.4 stages, p = 0.04) when completing the continuous, speed-graded, maximal effort exercise with versus without the soft robotic exosuit. Seven out of eleven (64%) study participants were able to train longer with the exosuit (see Table 2 and *Between-session differences in…duration* section). Six of these achieved a faster speed by progressing to a later exercise stage. Four of these six did so due to a delay in gait instability; two of these four eventually stopped due gait instability at a later stage, the third eventually stopped by self-request (i.e., gait instability was not observed), and the fourth was eventually stopped due to physiological signs that appeared at a later stage. The other two subjects able to progress to a later exercise stage with the exosuit did so due to a delayed onset of their original stopping criteria: one had delayed volitional fatigue and the other had delayed emergence of physiological signs requiring stopping. The seventh study participant who was able to train longer with exosuit assistance was able to do so due to an altered reason for stopping; during no-exosuit training they were stopped due to gait instability, whereas during exosuit-augmented training they trained until volitional fatigue. Altogether, five of the eight whose training duration during no-exosuit training was limited by gait instability demonstrated either a delay or absence of gait instability with the exosuit (Table 2).

**TABLE 2:** Summary of Session Stopping Reasons* and Final Stage Reached for the No-Exosuit and Exosuit Training Conditions

Participant	No-Exosuit Training	Exosuit Training
1	Gait instability, stage 6	Self, stage 7
2	Self, stage 6	Self, stage 8
3	Physiological, stage 2	Physiological, stage 7
4	Gait instability, stage 6	Gait instability, stage 7
5	Physiological, stage 2	Physiological, stage 2
6	Gait instability, stage 10	Gait instability, stage 11
7	Gait instability, stage 4	Physiological, stage 5
8	Gait instability, stage 4	Gait instability, stage 4
9	Gait instability, stage 5	Self, stage 5*
10	Gait instability, stage 7	Gait instability, stage 7
11	Gait instability, stage 5	Gait instability, stage 5

*. A continuous, speed-based, and graded exercise test (i.e., speed was progressively increased across stages) was administered for each condition under clinical supervision

^ɫ^The subject did not advance stages but trained longer at their final stage

### C. Between-Session Differences in Intensity and Duration

**Figure 3.**
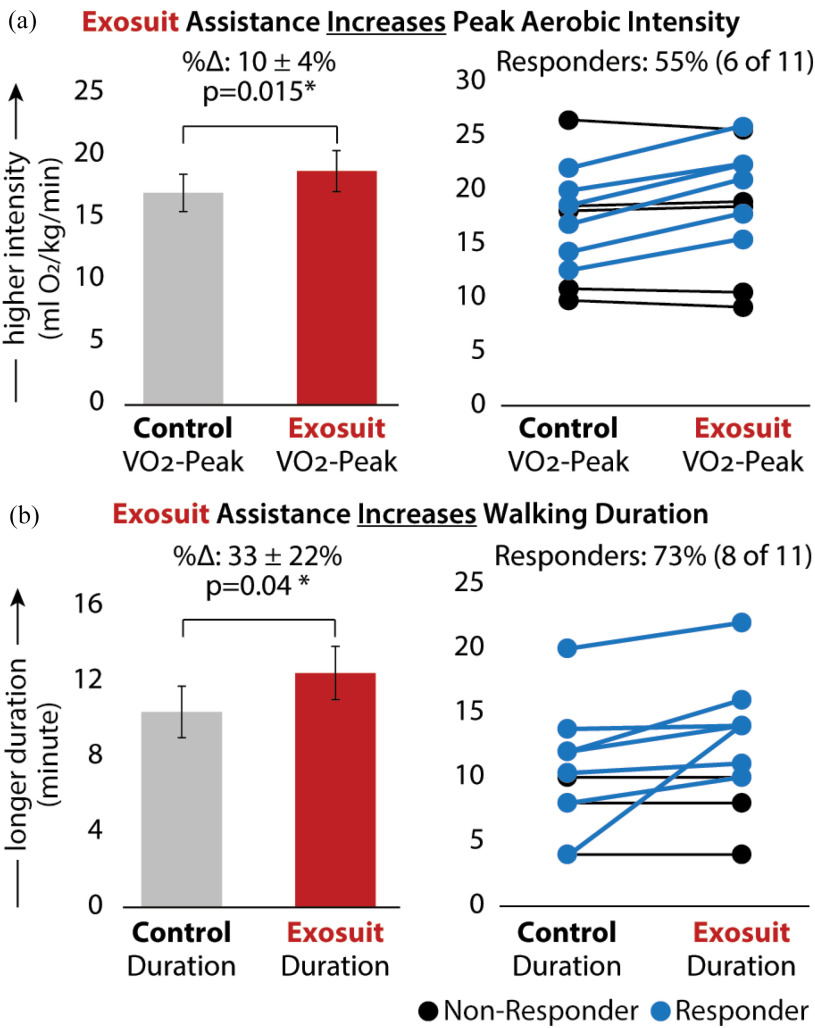
Between-condition comparison of training intensity and duration. Average (a) VO_2_ peak and (b) training duration for each training condition. The number of outcome-specific responders (blue) and non-responders (black) is shown to the right of each panel. VO_2_ responders were those with a between-condition (exosuit - control) increase in VO_2_ peak that was larger than the MDC (2.2 ml-O_2_/kg/min) (shown in blue). Training duration responders were those with a between-condition (exosuit – control) increase in duration of any magnitude. Significance (*) at p < 0.05.

The VO_2_ peak achieved during exosuit training was 10 ± 4% higher than in no-exosuit training (Δ: 1.75 ± 0.60 ml-O_2_/kg/min, p = 0.015; Fig. 3(a), left). Six out of eleven (64%) study participants increased their VO_2_ peak by at least the 2.2 ml-O_2_/kg/min minimal detectable change (MDC) score [36] (Fig. 3(a), right). These six responders had an average increase in VO_2_ peak of 3.39 ± 0.26 ml-O_2_/kg/min, compared to the negligible change observed in non-responders (Δ: -0.23 ± 0.58 ml-O_2_/kg/min).

Training duration was 33 ± 22% longer during exosuit training compared to no-exosuit training (Δ: 2.07 ± 0.88 min, p = 0.04; Fig. 3(b), left). Eight out of eleven (73%) study participants walked longer with exosuit assistance (average Δ: 2.84 ± 3 min), while the three others walked the same duration across training sessions (Fig. 3(b), right). No participant walked for a shorter duration with versus without exosuit assistance. Five of the six (83%) VO_2_ responders (shown in Fig. 3(a), right) also walked longer with exosuit assistance. The sixth responder walked for the same duration with and without exosuit-assistance.

### D. Training-Induced Changes in Serum BDNF

**Figure 4.**
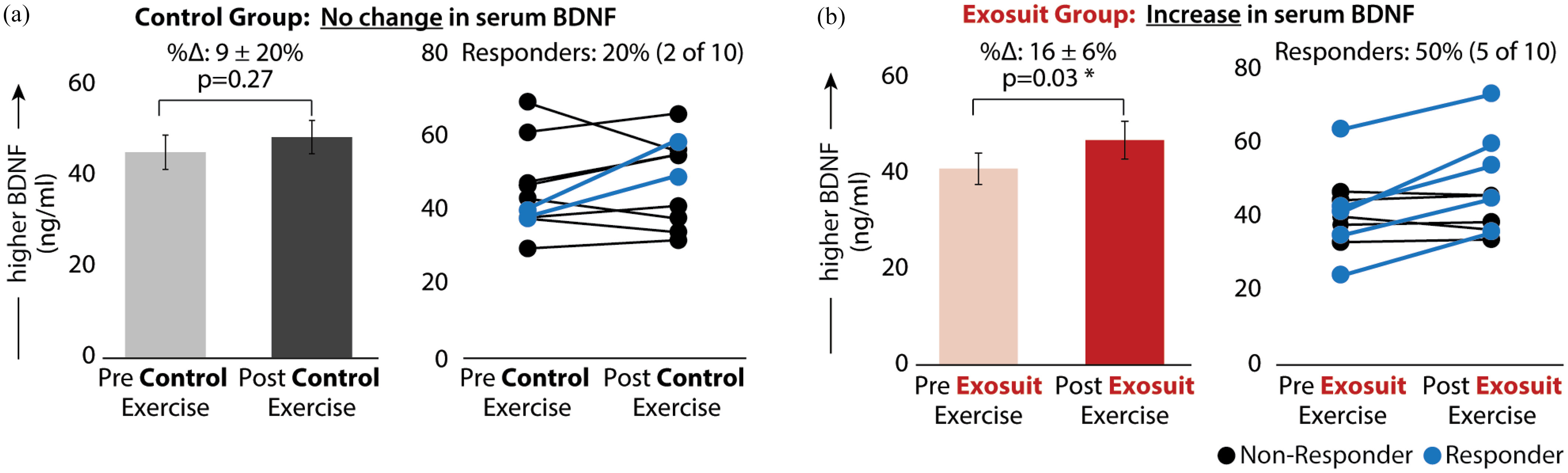
Serum BDNF levels pre-versus-post training. Average pre- and post-training serum BDNF levels in the (a) no-exosuit and (b) exosuit-augmented training conditions. The right of each panel also shows the number of BDNF responders—defined as those with a training-induced change in serum BDNF that exceeded the MDC-UCL_95_ (9.15 ng/ml) (shown in blue)—and non-responders (shown in black). Significance (*) at p < 0.05.

Post-training serum BDNF levels were significantly increased from pre-training levels after exosuit training (%Δ: 16 ± 6%, Δ: 5.96 ± 2.27 ng/ml, p = 0.03; Fig. 4(b), left). Five out of ten (50%) study participants increased BDNF levels by at least the MDC of 9.15 ng/ml [19]. These five responders had an average increase of 12.25 ± 1.62 ng/ml (Fig. 4(b), right). In contrast, post-training serum BDNF levels were not significantly different from pre-training levels after no-exosuit control training (%Δ: 9 ± 20%, Δ: 3.3 ± 2.86 ng/ml, p = 0.27; Fig. 4(a), left). Only two out of ten (20%) increased serum BDNF by the MDC. These two had an average increase of 14.8 ± 3.75 ng/ml (Fig. 4(a), right). Taken together, the average post-training serum BDNF level was 2.62 ng/ml higher following exosuit training compared to training without the exosuit.

### E. Training-Induced Changes in Propulsion Ability

**Figure 5.**
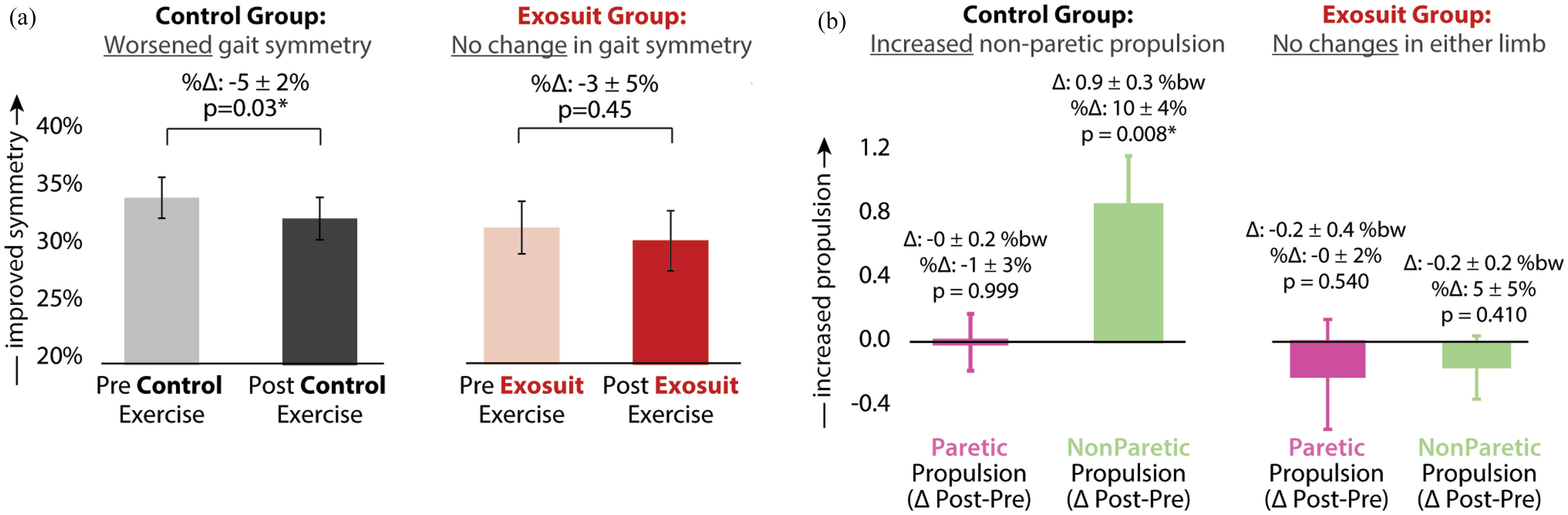
Propulsion symmetry pre-versus-post training. Average pre- and post-training (a) interlimb propulsion symmetry and (b) change in individual limb peak propulsion pre-to-post-training in the no-exosuit and exosuit-augmented training conditions. Significance (*) at p < 0.05 *Abbreviation*: %bw - %bodyweight.

Post-training propulsion symmetry was not significantly different from pre-training levels after exosuit training (Δ: -0.01 ± 0.01, %Δ: -3 ± 5%, p = 0.45; Fig. 5(a), right). In contrast, propulsion symmetry was significantly impaired after no-exosuit training (Δ: -0.02 ± 0.01, %Δ: -5 ± 2%, p = 0.03; Fig. 5(a), left). Inspection of changes in propulsion output from each limb revealed a significant increase in peak propulsion from the *non-paretic* limb after no-exosuit training (Δ: 0.9 ± 0.3 %bw, %Δ: 10 ± 4%, p = 0.008, Fig. 5(b), left). In contrast, significant changes in propulsion output were not observed for either the non-paretic or paretic limbs following exosuit training (p > 0.05) (Fig. 5(b), right).

## III. Discussion

We evaluated the effects of a soft robotic exosuit on training intensity and duration during continuous, speed-graded, maximal effort walking exercise. We hypothesized that exosuit-induced increases in aerobic intensity and training duration would be associated with a post-training increase in circulating BDNF levels and improvement in interlimb propulsion symmetry. Here we show that, compared to walking without exosuit assistance, soft robotic exosuits enable individuals with post-stroke hemiparesis to safely reach a higher VO_2_ peak by walking for a longer duration and faster speeds. We also observed significantly higher post-training BDNF levels following exosuit training only. Partially consistent with our hypothesis is our finding of no significant changes in propulsion symmetry following exosuit training, in contrast to a significant *worsening* in propulsion symmetry after no-exosuit training due to a significant increase in *non-paretic* peak propulsion.

### A. Between-Session Differences in Intensity and Duration

Our finding that soft robotic exosuit assistance facilitates faster treadmill walking, for a longer duration, and at a higher aerobic intensity contrasts with previous intervention studies of robotic-assisted walking after stroke. In fact the preponderance of evidence suggests that walking interventions performed with robotic exoskeletons do not improve functional walking outcomes of speed and distance [37]; the neuromuscular slacking measured while training in these devices is thought to be a possible reason [38]. Hence, clinical practice guidelines for walking recovery after stroke strongly recommend against robotic-assisted training to improve walking speed or distance in ambulatory individuals >6-mo post stroke [39].

Importantly, in most previous robot-assisted walking interventional studies after stroke, participants generally do *not* achieve targeted or prescribed aerobic intensities [40], [41]. Support for this idea lies in the work of Lefeber et al. who report reductions in physiological intensity measures (e.g., net minute ventilation, breathing frequency, perceived exertion, and heart rate) by nearly half during 30 minutes of robot-assisted compared to non-robot walking training (i.e., body-weight support treadmill training and over ground walking) [42]. While participants may in fact achieve high specificity and amount of walking practice during robotic interventions, a limited aerobic intensity during training may ultimately limit motor learning and recovery. This is because the intensity of training elicits bioenergetic changes that appear to signal neuroplastic mechanisms underlying experience-dependent learning [16]. In our work, we not only observed a significant average increase in VO_2_ peak, but also no significant differences (rather than significant reductions [42]) in additional physiological intensity metrics (see, *Supplementary Table III*). These results suggest that people post-stroke interact with a soft robotic exosuit differently than rigid robotic exoskeletons, and that this difference in human-robot interaction has different physiological consequences and rehabilitation implications.

An important consideration is that, in contrast to the full bodyweight support that many rigid exoskeletons provide, the soft robotic exosuit provides only partial assistance. This likely explains the physiological differences observed. That is, the partial assistance provided by soft robotic exosuits balances the necessary support of key gait subtasks (i.e., propulsion and ground clearance), while still requiring significant effort by the human body system(s) and/or musculature for movement, versus the device's mechanical support. Future work investigating the underlying muscular response to soft robotic exosuit assistance is critical for understanding human-exosuit interaction in the context of our observed physiological responses.

### B. Training-Induced Changes in Serum BDNF

The average increase in serum BDNF observed in this study was similar to the average post-acute exercise change reported in the post-stroke literature (2.49, 95% CI 1.10, 3.88) [20]. Our findings are thus consistent with the idea that peripheral BDNF levels increase in response to an acute bout of high intensity exercise in people after stroke [19], [20], with this study showing that this effect can be enhanced by interventions that enable training at a higher intensity and duration—such as the soft robotic exosuit. Reasons for the observed inter-individual variability include training dosing, factors influencing energy balance (e.g., nutrition, stress), genetic polymorphisms (e.g., Val66Met), and tissue crosstalk (discussed further in *Supplementary Materials*).

### C. Training-Induced Changes in Peak Propulsion Symmetry

The increase in propulsion asymmetry observed after no-exosuit training, but not exosuit-augmented training, suggests that fast walking training without the biomechanical support of a soft robotic exosuit may result in people post-stroke increasing their compensatory reliance on the non-paretic limb for propulsion [43]. Indeed, in addition to increasing the overall propulsive demands of walking, the speed-graded, maximal effort training session may have resulted in fatigue. Together, increased propulsive demands and fatigue may have led to increased gait compensation. That is, it is plausible that during the no-exosuit training session, subjects relied primarily on increasing non-paretic limb propulsion to achieve the fast walking speeds required, and this compensation was immediately retained and demonstrated during post-training testing. To confirm this hypothesis, future studies must characterize the effects of exosuit assistance *during* training. Nonetheless, these findings suggest that exosuit assistance may be harnessed by the user to prevent a worsening of propulsion symmetry during fast walking.

### D. Clinical Impact

Soft robotic exosuits may be a clinically practical approach to maximizing intensity and promoting intensity-dependent BDNF release *during* walking training. An alternative approach to leveraging exercise-induced increases in circulating BDNF to enhance motor learning during walking training is to provide a bout of high intensity exercise (e.g., using a stationary bike, full body stepper, or treadmill) *before* or *after* walking training. Indeed, several motor learning paradigms have leveraged a bout of high intensity exercise to prime learning before the learning task [5], [6], [9]. However, this approach is not clinically practical. The ∼20 minutes of high intensity “pre-” training that may be required to induce significant changes in serum BDNF levels [19] take away from the already limited time available for walking practice and will also mentally and physically fatigue the majority of patients before they even begin walking training. For most of our participants (63%), exosuit assistance delayed the onset of gait instability or physiological signs of distress, enabling these participants to walk until later stages and reach faster speeds. For the participants in this study, exosuit-assisted walking was at least as safe as fast walking without exosuit assistance. Exosuits have the potential to facilitate safer walking at a higher aerobic intensity and longer training duration than gait training without exosuits. The potential for exosuits to enable more effective walking rehabilitation by way of facilitating walking-specific practice of greater intensity and repetition—salient training parameters for inducing experience-dependent neuroplasticity [34]—warrants study.

#### 1) Limitations

A primary limitation of this study is the small sample size of high functioning, community-dwelling individuals poststroke. A larger, more diverse sample of post-stroke individuals is needed to fully characterize the therapeutic value of soft robotic exosuits. Moreover, given the sample size, we were not powered to examine between-group differences in the BDNF and peak propulsion symmetry outcomes. In addition, because the commercial ReStore exosuit does not provide access to the raw IMU data, the study did not include biomechanical measurements *during* training, and is thus unable to evaluate how users’ biomechanical interaction with the exosuit may relate to the study's outcomes. Other methodological limitations of this study include the lack of genotyping for the single nucleotide polymorphism, Val66Met, on the BDNF gene [44] as the presence of Val66Met [44] appears to alter BDNF processing and cell signaling, leading to modified activity-dependent release of BDNF [45].

## IV. Conclusion

Individuals with chronic post-stroke hemiparesis safely achieve faster treadmill training speeds, a longer training duration, and higher training intensity when completing continuous, speed-graded walking training with versus without a soft robotic exosuit. These findings, taken together with the higher post-training BDNF levels observed after exosuit-augmented training (but not control training), highlight the rehabilitative potential of exosuit-augmented, high intensity gait training. Moreover, soft robotic exosuits appear to mitigate an increased reliance on non-paretic limb propulsion compensation when people post-stroke train at fast walking speeds. This foundational study provides clinical, physiological, and biomechanical evidence in support of using soft robotic exosuits to provide more effective post-stroke gait rehabilitation in appropriate users. Future study of the effects of multiple sessions of high intensity, robotic exosuit-augmented locomotion is warranted to fully elucidate the therapeutic potential of soft robotic exosuits applied as rehabilitation technology.

## V. Materials and Methods

Please refer to the *Supplementary Materials* for a detailed description of data collection, processing, and statistical analysis procedures.

### A. Participants

*Inclusion criteria:* diagnosis of stroke >6 months, 18–80 years old, independent ambulation without the support of another individual for at least 2 minutes (assistive device allowed), and medical clearance by a physician. Exclusion criteria: inability to clearly communicate with research staff; serious musculoskeletal, cardiovascular, pulmonary, or neurological (other than stroke) comorbidities that may interfere with ability to participate; self-reported anemia (hemoglobin levels of <13 g/dL for men and <12 g/dL for women); untreated clotting disorders; unexplained dizziness in prior months; donated blood ≤60 days; unresolved deep vein thrombosis, uncontrolled or untreated hypertension; ankle dorsiflexion range of motion limited to 5° plantarflexion; open wound at device location; urethane allergies; and pregnancy. All participants were consented according to the study protocol approved by Boston University's Institutional Review Board.

### B. Continuous, Speed-Graded, Max Effort Training Protocol

Each training session consisted of a continuous, speed-graded, maximal effort exercise bout that followed the same progression protocol: initial treadmill testing speed was set to 80% of comfortable overground walking speed and tested for 2 minutes, followed by 10% increases in walking speed every 2 minutes until walking was stopped. Stopping criteria included reaching peak volitional fatigue or meeting any physiological or physical stopping criteria. Stopping criteria were based on previously published maximal effort exercise testing standards (see [46] and *Supplementary Table I*). Participants were harnessed for safety during walking and guarded by a physical therapist; no bodyweight was supported. Training sessions took place at least three days but no more than 2 weeks apart at the same time of day [47]. Participants abstained from alcohol for 24 hours, caffeine for 12 hours, and fasted for at least 3 hours prior to exercise.
